# Oral implant osseointegration model in C57Bl/6 mice: microtomographic, histological, histomorphometric and molecular characterization

**DOI:** 10.1590/1678-7757-2017-0601

**Published:** 2018-05-22

**Authors:** Claudia Cristina Biguetti, Franco Cavalla, Elcia M. Silveira, Angélica Cristina Fonseca, Andreia Espindola Vieira, Andre Petenuci Tabanez, Danieli C. Rodrigues, Ana Paula Favaro Trombone, Gustavo Pompermaier Garlet

**Affiliations:** 1 Universidade de São Paulo, Faculdade de Odontologia de Bauru, Departamento de Ciências Biológicas, Bauru, São Paulo, Brasil.; 2 Universidad de Chile, Facultad de Odontología, Departamento de Odontología Conservadora, Santiago, Chile.; 3 Universidade do Sagrado Coração, Departamento de Ciências Biológicas e da Saúde, Bauru, Brasil.; 4 Universidade Federal de Alagoas, Instituto de Ciências Biológicas e da Saúde, Alagoas, Brasil.; 5 University of Texas at Dallas, Department of Bioengineering, Dallas, Texas, United States.

**Keywords:** Osseointegration, Dental implants, Peri-implant endosseous healing, Bone implant interface

## Abstract

Despite the successful clinical application of titanium (Ti) as a biomaterial, the exact cellular and molecular mechanisms responsible for Ti osseointegration remains unclear, especially because of the limited methodological tools available in this field.

**Objective:**

In this study, we present a microscopic and molecular characterization of an oral implant osseointegration model using C57Bl/6 mice.

**Material and Methods:**

Forty-eight male wild-type mice received a Ti implant on the edentulous alveolar crest and the peri-implant sites were evaluated through microscopic (μCT, histological and birefringence) and molecular (RealTimePCRarray) analysis in different points in time after surgery (3, 7, 14 and 21 days).

**Results:**

The early stages of osseointegration were marked by an increased expression of growth factors and MSC markers. Subsequently, a provisional granulation tissue was formed, with high expression of VEGFb and earlier osteogenic markers (BMPs, ALP and Runx2). The immune/inflammatory phase was evidenced by an increased density of inflammatory cells, and high expression of cytokines (TNF, IL6, IL1) chemokines (CXCL3, CCL2, CCL5 and CXC3CL1) and chemokine receptors (CCR2 and CCR5). Also, iNOS expression remained low, while ARG1 was upregulated, indicating predominance of a M2-type response. At later points in time, the bone matrix density and volume were increased, in agreement with a high expression of Col1a1 and Col21a2. The remodelling process was marked by peaks of MMPs, RANKL and OPG expression at 14 days, and an increased density of osteoclasts. At 21 days, intimate Ti/bone contact was observed, with expression of final osteoblast differentiation markers (PHEX, SOST), as well as red spectrum collagen fibers.

**Conclusions:**

This study demonstrated a unique molecular view of oral osseointegration kinetics in C57Bl/6 mice, evidencing potential elements responsible for orchestrating cell migration, proliferation, ECM deposition and maturation, angiogenesis, bone formation and remodeling at the bone-implant interface in parallel with a novel microscopic analysis.

## Introduction

titanium (Ti) is considered the gold standard biomaterial in oral implantology^1^, due to the material’s high biocompatibility, adequate mechanical properties, and osseointegration capacity^1^ ^,^ ^2^, which lead to long-term performance and high rates of clinical success^1^ ^,^ ^3^. Additionally, Ti is also currently regarded as an immunomodulatory biomaterial rather than an inert metal, since Ti implantation in bone is associated with a transitory small degree of inflammation, which seems to contribute to the activation of host pathways that leads to osseointegration^2^ ^,^ ^4^. However, despite the clinical success and widespread application of Ti-based devices in Dentistry and Medicine, the exact cellular and molecular mechanisms responsible for the osseointegration phenomenon remains unclear^4^, especially considering the immunological pathways involved in this process.

Most studies in the field of osseointegration have focused on surface modifications of Ti and their possible impact on the bone apposition outcome^5^. Indeed, most *in vitro* studies have focused on the surface topography and surface chemical composition of Ti with different treatments and coatings, aiming at the improvement of bone cells differentiation and matrix apposition/mineralization^5^. While useful in several aspects, *in vitro* studies are limited due to the intrinsic characteristics of cell culture, which evidently does not simulate all the biomaterial-host tissue interactions that take place *in vivo* ^6^. In addition, *in vivo* preclinical evaluation of bone formation and remodelling on Ti surfaces are usually performed in animals with robust skeletal bones, such as minipigs^7^ and dogs^8^, which can recapitulate the architecture of human craniofacial bones and allow the analysis of implant modification (i.e. shapes, coatings and/or surface topographies) in osseointegration^9^. While such large animal-based models are useful for certain applications, inherent factors such as animal size/weight, lack of specific experimental tools for cause-and-effect experiments, as well as absent or restricted molecular assays, limit the possibilities of understanding the biological basis of osseointegration. In this scenario, mice have been demonstrated to be a suitable animal model to properly investigate cellular and molecular aspects of a series of biological processes due to a number of experimental tools available for dissecting biological mechanisms^9^.

Mouse models have several advantages including: 99% similarity to the human genome; availability of a number of efficient genetic/molecular tools; the animal’s small size facilitates the use of reduced quantities of drugs and reduced experimental periods, making it a cost-efficient model^11^. Additionally, there is a large availability of wild-type strains with distinct host response features, as well numerous genetically engineered mice strains, particularly with the C57Bl/6 background^11^. Consequently, such model allows valuable cause-and-effect experimentation to determine gene/cell functions in bioengineering and regenerative processes^9^ ^,^ ^12^.

Finally, the use of mice in the Osteoimmunology field as an experimental model host results in additional advantages due to the extensive knowledge on the inflammatory and immunological responses of mice^9^ ^,^ ^13^. In this context, endochondral long bones osseointegration models have been developed in mice with different approaches, such as for investigation of molecular and cellular regulation of osseointegration under micromotion stimuli^15^, implant stability and insertion torque^16^, and acceleration of osseointegration^17^. In this context, osseointegration in long/endochondral bones is achieved through the program of endochondral ossification, which differs from osseointegration in the maxillary/mandibular bone. In addition, there is a large proportion of marrow cavity in the implantation sites of long bones, which exhibit the slowest reaction to implant placement compared to the periosteum region^16^. Therefore, while these studies are useful to better understand the osseointegration process in orthopaedics applications, they cannot be fully translated for the Dentistry (i.e. maxillary/mandibular implants) context.

On the other hand, maxillary and mandibular intramembranous bones are characterized by distinctive functional, anatomical and embryological features when compared to long bones, which could result in different aspects in the outcome of bone repair during osseointegration^3^. Thus, two different mice strains have been used in oral osseointegration studies: CD1 and C57/Bl-6 mice. Using CD1 mice strain, oral osseointegration models have been developed in the edentulous alveolar crest in front of the first maxillary molar^17^ or by using a healed alveolar socket after extraction of the upper molars^28^. However, despite the advantage of having a robust skeletal phenotype compared to other mice strains, CD1 is an outbreed strain, which adds some genetic variability as a limitation to this model, and also limits its genetic manipulation^26^. Alternatively, the use of C57Bl/6 mice overcomes some of these limitations, since this inbred strain have a widely known genetic background^18^, being the mostly used strain in immunological studies^19^. However, the C57Bl/6 mice oral osseointegration model has been used for studying microtomographic and histological aspects of peri-implantitis^28^, by focusing on late stages of osseointegration and not on the entire bone repair process by which osseointegration is achieved.

Therefore, in this study we propose to combine the advantages of previously developed models, using the edentulous alveolar crest (avoiding the limitations and complications of tooth extraction requirements) of C57Bl/6 mice (supported by the extensive knowledge and additional experimental possibilities inherent to this strain) as the implant placement site, followed by a detailed microtomographic, histological, histomorphometric, and molecular characterization of the osseointegration process.

## Material and methods

### Animals

Forty-eight male wild-type mice (C57Bl/6) (10 weeks old, 25 g of weight on average) were obtained from the animal facilities of FOB/USP. Thirty-six animals were used for microscopic analysis (microCT, histological, and birefringence analysis) and twelve animals were used for molecular assays, distributed along 4 experimental periods: 3, 7, 14 and 21 days after surgical procedure. Throughout all experimental periods of this study, the mice were provided sterile water *ad libitum* and were fed with sterile standard solid mice chow (Nuvital, Curitiba, PR, Brazil), except during the first 72 hours after surgery, in which their diet was crumbles. No antibiotics and anti-inflammatory drugs were administered to the animals after implantation surgery and there was no evidence of weight loss, infection and persistent inflammation in the surgical sites. This study was carried out in strict accordance with the recommendations of the Guide for the Care and Use of Laboratory Animals of the National Institutes of Health^20^, and the experimental protocol was approved by the local Institutional Committee for Animal Care and Use (#012/2014).

### Titanium implant screws

In an attempt to employ a titanium screw comparable to the one clinically used in Dentistry, a screw with Ø 0.6 mm, titanium-6 aluminum-4 vanadium alloy (NTI-Kahla GmbH Rotary Dental Instruments, Kahla, Thüringen, Germany) and machined titanium surface was used in this study, as previously described in the oral osseointegration model in CD1 mice^10^. The screws were cut at a length of 1.5 mm and sterilized by autoclaving before surgical procedures. Subsequently, the screws were analyzed via scanning electron microscopy (SEM) and energy dispersive X-ray (EDX) before Ti implantation, in order to demonstrate the surface topography and chemical composition of the screws used in this study. The screws were fixed on SEM-stub-holders and imaged with an ultra-high resolution SEM (FEI Nova NanoSEM, Thermo Fisher Scientific, OR, USA) at 8kV with a resolution of 127.8 eV. The chemical composition was analyzed in the same regions of interest for qualitative SEM images, by using the software TEAM™ EDS Analysis System (AMETEK Materials Analysis Division, Mahwah, NJ, USA) in relation to the amount of 10 chemical elements present in the bulk structure of clinically used titanium implants, as previously described^21^: Titanium (Ti), Aluminum (Al), Vanadium (V), Calcium (Ca), Nitrogen (N), Niobium (Nb), Oxygen (O), Phosphorus (P), Sulfur (S) and Zinc (Zn).

### Experimental protocol

**Figure 1:**
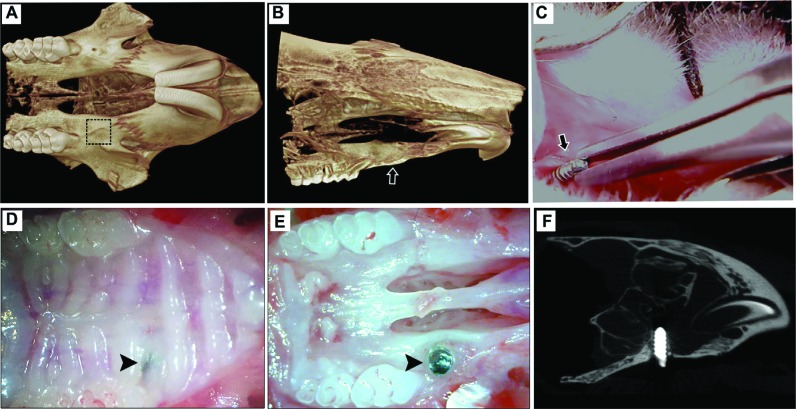
Experimental protocol for oral osseointegration in C57Bl/6 mice. A-B) Microtomographic tridimensional images from mouse maxilla bones showing the area of interest for screw implantation between the maxillary right first molar and the incisor (A-dotted square, B arrow); C) Ti-screw was screwed down in the implant bed (arrow), using a castroviejo Micro Needle Holder (Fine Science Tools^®^, British Columbia, CA); D) Macroscopic clinical view from oral mucosa covering the Ti-screw (arrow head) after day 7 post implantation and E) the same specimen without soft tissues post euthanasia; F) Representative microtomographic sagital slice from mouse maxilla at day 7 post Ti screw implantation

Before the surgical procedure, microtomographic images of three different mouse maxillae were carefully measured considering thicker areas to install the titanium implants, which had 300 µm of thickness, between the maxillary right first molar and the incisors (Figure 1AB). For the surgical procedure, the mice were anesthetized through intramuscular administration of 80 mg/kg of ketamine chloride (Dopalen^®^, Agribrands Brasil LTDA, Paulínia, SP, Brazil) and 160 mg/kg of xylazine chloride (Anasedan^®^, Agribrands Brasil LTDA, Paulínia, SP, Brazil) in a 1:1 proportion, which was determined according to the animal’s weight. Subsequently, the mice were placed on a surgical table with a mouth retractor, as previously described in other Dentistry mice models^14^ ^,^ ^22^. Oral titanium implant screws were placed in the C57Bl/6 mice following a previous surgical protocol described for CD1 mice^10^, and each mouse received one oral implant inserted in the left edentulous alveolar crest. Oral mucosa was cleaned using a topical chlorhexidine solution for 1 min followed by an incision with 2 mm width parallel to the palatal crease and 1 mm in front of the left first maxillary molar, by using a 22.5° angled micro scalpel blade (n.10316-14, Fine Science Tools^®^, British Columbia, CA, USA). A small detachment of the mucoperiosteum was made and the subjacent bone was drilled using a pilot drill with Ø 0.50 mm (NTI-Kahla GmbH Rotary Dental Instruments, Kahla, Thüringen, Germany). The pilot hole was performed using a surgical motor (NSK-Nakanishi International, Kanuma, Tochigi, Japan), with 600 rpm speed and 35 N to kilogram force, under continuous irrigation with cold saline solution, in order to avoid heating and subsequent bone necrosis. The Ti-implant was screwed down in the implant bed using a Castro Viejo Micro Needle Holder (Fine Science Tools^®^, British Columbia, CA, USA) (Figure 1C). All surgical procedures were performed by a single calibrated surgeon (FC). At the end of the experimental periods (days 3, 7, 14 and 21 post-Ti screw implantation), the mice were killed with an excessive dose of anesthetic and the maxillae were collected. Nine maxillae were used for microscopic [micro-computed tomography (μCT), histological and birefringence] analyses; and three samples containing only the region of the implant bed were used for RealTimePCRarray analysis. The samples designated for microscopic analysis were fixed in PBS-buffered formalin (10%) solution (pH 7.2) for 48h at room temperature, subsequently washed over-night in running water and maintained temporarily in alcohol fixative (70% hydrous ethanol) until the conclusion of the μCT analysis, and then decalcified in 4.13% EDTA (pH 7,2). After the samples’ decalcification, the Ti screw was carefully unscrewed from the implant bed with a Micro Needle Holder for histological processing and paraffin inclusion. The samples for molecular analysis were stored in RNAlater (Ambion, Austin, TX, USA) solutions^9^.

### Micro-computed tomography (μCT) assessment

Thirty-six mouse maxillae containing the Ti-implants were scanned with the Skyscan 1176 System (Bruker Microct, Kontich, Belgium) at 80 kV, 300 μA, 180 degrees of rotation and 1-degree exposure range. The images were captured with a 12.45 μm pixel size resolution. The projection images were reconstructed using the NRecon software (Bruker Microct, Kontich, Belgium) with 35% of Beam Hardening Correction and subsequently aligned using the Dataviewer 1.4.4.0 software (Bruker Microct, Kontich, Belgium) to standardize the position of all specimens for subsequent quantitative evaluation (Figure 3A-C). The three-dimensional images obtained were analyzed with the CT-Vox 2.3 software. The quantitative evaluation of bone to implant interface was carried out using the CTAn 1.1.4.1 software (Bruker Microct, Kontich, Belgium) in accordance with the recommended guidelines^23^. Briefly, for measuring the proportion of bone volume (BV/TV, %) at the implant-bone interface area, the data set of images saved in axial position was opened in the CTAn software and the region of interest (ROI) was determined using a cylindrical segmentation with 500 µm axis length and 700 µm diameter(Figure 3C). The first 200 μm from the first third of the Ti screw were excluded from the ROI to standardize the positioning for the starting bone quantification in all specimens, as demonstrated in Figure 3B. The bone quantification was performed considering 100 µm from the implant surface in an axial view, into the bone (Figure 3C). After binarization and separation between titanium body and bone through the difference of hyperdensities, the BV/TV was acquired (Figure 3E).

### Histomorphometry

The same mice maxillae used for microCT scanning were processed for histological analysis. Forty semi-serial sections were cut with 4 µm thickness, of which nine serial sections considering the central region of bone for implant contact were chosen for histomorphometry and stained for hematoxylin and eosin (H&E) staining. The analyses were performed by a single calibrated investigator with a binocular microscope (Olympus Optical Co., Tokyo, Honshu, Japan) using a 100x immersion objective. Six histological fields *per* HE section, comprising the region adjacent to the thread spaces, were captured using a 100× immersion objective. A grid image was superimposed over each histological field, with 10 parallel lines and 100 points in a quadrangular area, by using the Image J software (Version 1.51, National Institutes of Health, Bethesda, MD, USA). Briefly, the points were counted coinciding with the following parameters of the osseointegration process: blood clot, inflammatory cells, other elements (empty spaces left by the implant’s space), blood vessels, fibroblasts, collagen fibers, osteoblasts, osteoclasts, and new bone matrix. The results were presented as the mean area density for each structure considered in each examined group.

### Picrosirius-polarization method and quantification of birefringent fibers

For birefringence analysis, 4 sections with 5 µm thickness histological slides considering the central region of the bone for implant contact were used for picrosirius red staining and birefringence analysis. As previously described^9^, green birefringence color indicates thin fibers; yellow and red colors at birefringence analysis indicate thick collagen fibers. Three fields from each section were analyzed through polarizing lens coupled to a binocular inverted microscope (Leica DM IRB/E, Leica Microsystems Wetzlar GmbH, Wetzlar, Germany), by using 40x magnification immersion objective. All the images were captured with the same parameters (the same light intensity and angle of the polarizing lens at 90° from the light source) from the Leica Imaging Software (LAX, Leica Microsystems Wetzlar GmbH, Wetzlar, Germany). Briefly, the quantification of birefringence brightness was performed using software AxioVision 4.8 (Carl Zeiss Microscopy GmbH, Jena, Germany). The images were binarized for green, yellow and red spectra, and the quantity of each color pixels^2^ corresponding to the total area of each histological field was measured^9^. Mean values corresponding to 4 sections from each animal were calculated in pixels^2^.

### RealTimePCR array reactions

The samples containing only the region of the implant bed were resected and stored in a RNA Stabilization Solution (RNAlater^®^, Thermofisher, Waltham, MA, USA) until the RealTime PCR array reactions. The RealTimePCR array reactions were performed as previously described^9^ ^,^ ^24^ ^,^ ^25^. First, the RealTimePCR array was performed from a pool of all experimental time-points (3 d, 7 d, 14 d and 21 d), providing targets in which expression variation was significant compared to the control side. Then, upregulated targets were analyzed regarding their kinetics of expression for the specific time points of 3, 7, 14 and 21 days during the osseointegration process. Briefly, the extraction of total RNA from the implantation site was performed with a RNeasyFFPE kit (Qiagen Inc, Valencia, CA, USA) according to the manufacturers’ instructions. The integrity of the RNA samples was checked by analyzing 1 mg of the total RNA with 2100Bioanalyzer (Agilent Technologies, Santa Clara, CA, USA) according to the manufacturers’ instructions, and the complementary DNA was synthesized using 3 µg of RNA through a reverse transcription reaction (Superscript III, Invitrogen Corporation, Carlsbad, CA, USA). The Real-time PCR array was performed in a Viia7 instrument (LifeTechnologies, Carlsbad, CA, USA) using custom panels for “wound healing” (PAMM-121), “inflammatory cytokines and receptors” (PAMM-011) and “osteogenesis” (PAMM-026) (SABiosciences, Frederick, MD, USA) for gene expression profiling. Data were analyzed using the RT2 Profiler PCR Array Data Analysis online software (SABiosciences, Frederick, MD, USA) for normalizing the initial geometric mean of three constitutive genes (GAPDH, ACTB, Hprt1), following the normalizing of the control group. Data are expressed as heat map fold change relative to the control group.

### Statistical analysis

Differences between data sets were statistically analyzed through One-Way Analysis of Variance (ANOVA) followed by Bonferroni’s multiple comparison *post-hoc* test or student’s t-test where applicable; for data that did not fit in the distribution of normality, Kruskal-Wallis test (followed by Dunn’s test) and Mann-Whitney test were used. The statistical significance of the experiment involving the PCR Array was evaluated through the Mann-Whitney test, and the values were tested for correction of Benjamini and Hochberg^26^ (1995). Values of p<0.05 were considered statistically significant. All statistical tests were performed with the GraphPad Prism 5.0 software (GraphPad Software Inc., San Diego, CA, USA).

## Results

### Development of the surgical protocol

Our focus is this study was to address a pre-clinic murine model of oral osseointegration, previously developed in CD1 mice^10^ for C57Bl/6 mice. We first analyzed the anatomy of three different maxillae from 10-week C57Bl/6 male mice, through microtomographic images, and then selected the most robust skeletal area as an implant bed, specifically in the edentulous space between the maxillary right first molar and the incisor, along the alveolar crest, comprising an average 300 µm of thickness (Figure 1AB).

**Figure 2:**
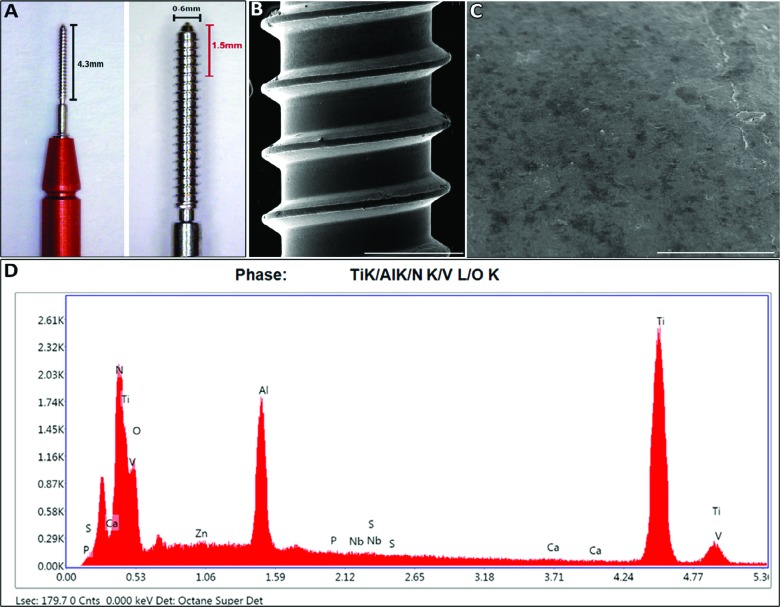
Ti screw used in oral osseintegration model in C57Bl/6 mice. A) Ti-screw (NTI-Kahla GmbH Rotary Dental Instruments, Kahla, Thüringen, Germany) of Ø0.6 mm was cut at length of 1.5 mm; B) Surface morphology of the body of titanium screw (385x magnification, scale bar 300 µm) and its (C) machined surface topography (5225x, scale bar 20 µm) from scanning electron microscopy (SEM) micrograph; D) Representative graph with surface composition from EDX analysis

SEM micrographs demonstrated uniform unidirectional threads, with no deposits and no particular features or deformation and characteristics of a clearly machined surface topography, such as small irregularities. In the composition characterization, Ti screw alloy had a mass with 75.35% of Ti, 14.66 % of V, 5% of N and 4.20% of Al. Other evaluated chemical elements were found in minor concentration, less than 1% (Figure 2D).

For developing the surgical protocol, the Ti-screw was implanted in the edentulous space between the maxillary right first molar and the incisor. After a day of surgery, the animals were able to eat crumbled food and were acting normally, with no signs of distress. All animals had complete oral mucosal healing by day 7, as clinically demonstrated in Figure 1D. It is important to note that of the 36 implants placed and investigated with microscopy, 33 demonstrated primary stability immediately after the screw’s insertion and 28 achieved osseointegration, observed through microCT and histologic assessment, and totaling a 77.78% success rate in terms of osseointegration. Additionally, the 5 implants which exhibited failure after 14 and 21 days, did not show signs of infection in the histological and clinical examination.

### μCT assessment

**Figure 3:**
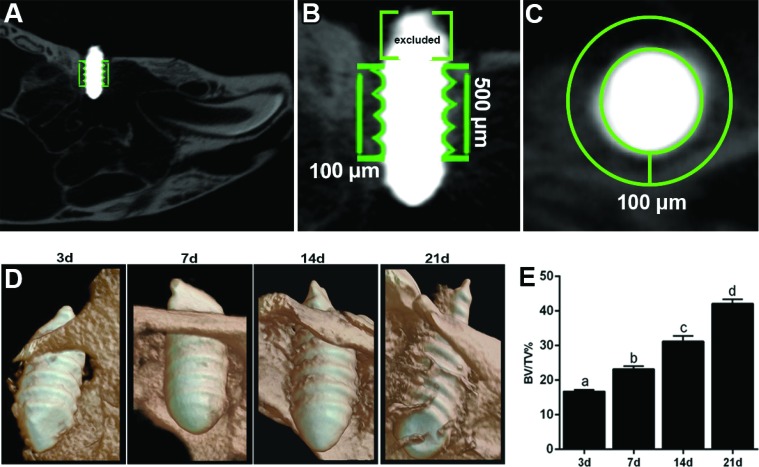
Micro-computed tomography (µCT) analysis of oral osseointegration model in C57Bl/6 mice. A) 2-D sagittal view of maxilla containing Ti screw for bone quantification; B) 2-D sagittal view with delimitation of a region of interest in the contact area of bone-to-implant, covering the region of contact bone threads throughout 500 µm of implant body and in the interface between the threads. The first 200 µm from the first third of Ti screw was excluded analysis in order to standardize the positioning for starting bone quantification in all specimens; (C) Axial view of Ti screw and bone inside the region of interest, considering 100 µm from the implant surface into the bone; D) Three-dimensional images were obtained with the CT-Vox software (Bruker Microct, Kontich, Belgium) along 3,7,14 and 21 days along osseointegration; E) Proportion of bone volume/tissue volume (BV/TV, %) in the interface bone-Ti were evaluated using CTAn software (Bruker Microct, Kontich, Belgium) to measure along days 3, 7, 14 and 21 post implantation. Different letters indicate significant statistical differences (p<0.05) among time periods

Subsequently, we evaluated the sites of Ti-implantation through microtomographic qualitative and quantitative analyses of mineralized bone matrix (Figure 3A-C). The three-dimensional images of maxillae containing the sites of the Ti implants (Figure 3D), as well as the quantitative assessment (BV/TV) indicated gradual and significant bone apposition (BV/TV, %) around the implant threads throughout 7 d (23.19±2.014), 14 d (31.20±3.82) and 21 d (42.12±3.01) (Figure 3E). After 3 days, the bone detected through microCT (16.73±1.11) was predominantly comprised by the native/remaining bone supporting the Ti-screw, as demonstrated by the representative three-dimensional image (Figure 3D). The newly formed bone matrix was detected 7 days after implantation, as evidenced by Figure 3E. The maximum amount of osseointegration was achieved after 21 days, when the bone/Ti interface was covered with an average 42.12±3.01% of BV/TV (Figure 3E).

### Histology, histomorphometry and birefringence

**Figure 4:**
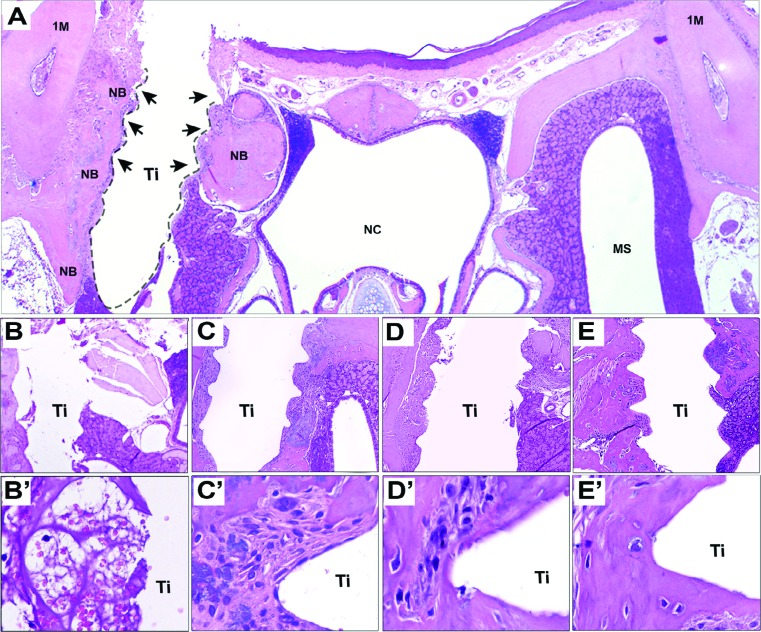
Hematoxylin & eosin (HE) staining of oral osseointegration model in C57Bl/6 mice and its histological aspects. A) Representative panoramic section of mouse maxilla and region of Ti implantation at day 14 post surgery. Arrows show threads space in direct contact with newly formed bone (NB); B-E’) Chronology of oral osseointegration is observed throughout days 3 (B10x, B’40x), 7 (C10x, C’40x), 14 (D10x, D’40x) and 21 (E10x, E’40x). HE staining. NB= Newly formed bone. Ti= Ti screw space. 1M= first molar. NC= Nasal Cavity. MS= Maxillary sinus

**Figure 5:**
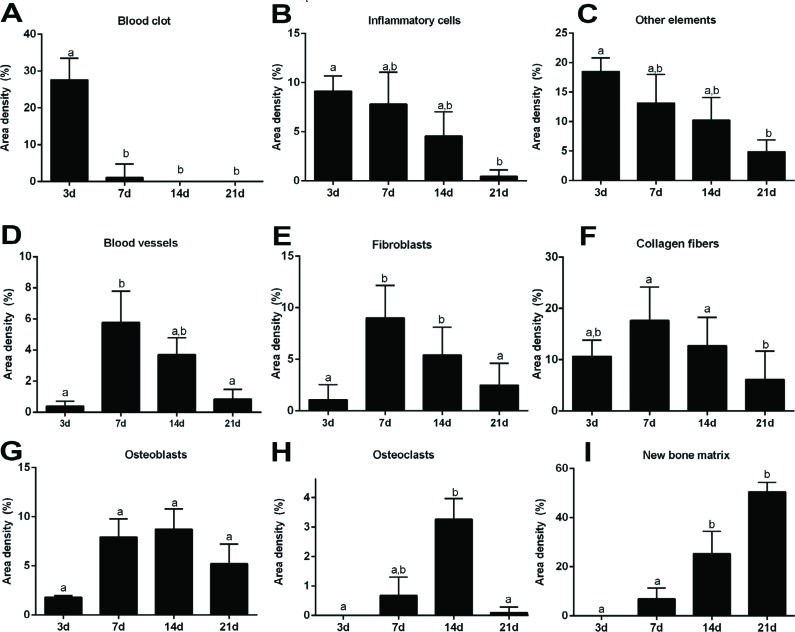
Histomorphometric analysis of healing components along oral osseintegration process in C57Bl/6-WT mice. Results are presented as the means (±SD) of area density for each component related to osseointegration process: (A) Blood clot; (B) Inflammatory cells; (C) Other elements; (D) Blood vessels; (E) Fibroblasts; (F) Collagen fibers; (G) Osteoblasts; (H) Osteoclasts; (I) New bone matrix. Different letters indicate a statistically significant difference between the different time periods (p<0.05)

Considering the histological analysis, the panoramic transversal image of mouse maxillae demonstrated that the Ti-screw was projected through the palatal bone into the olfactory epithelium of the maxillary sinus (histological section after 14 days, Figure 4A), as also described previously in CD1 mice^10^. The histological and histomorphometric analysis were performed in the spaces occupied by the three initial Ti-screw threads, from coronal to apical, on each side of the Ti-screw, as indicated by arrows in Figure 4A. After 3 days, the bone-implant interface was filled mainly by a blood clot and inflammatory infiltrate, as demonstrated through histomorphometry (Figure 5A, B). The blood clot was evidenced by erythrocytes, surrounded by an eosinophilic and slight matrix of the fibrin network, also permeated by an inflammatory infiltrate with predominance of mononuclear cells (Figure 4B and B’). It is important to note that there was no newly formed bone matrix after 3 days. Consequently, the bone matrix quantified after 3 days was merely native viable bone and bone debris observed around the Ti threads.

At the 7^th^ day after implantation, there was a significant decrease in the blood clot (Figure 5A), while the area density of fibroblasts and blood vessels significantly increased (Figure 5D, E), as a consequence of the formation of a transitory granulation tissue (Figure 4C and C’). Aligned robust and cuboid cells, with a typical morphology of osteoblasts, were also observed producing new bone matrix between the implant surface and pre-existing bone. Also after 7 days, osteoclastic resorption lacunae and a small number of osteoclasts were found around the bone debris and pre-existing bone. From 14 to 21 days, granulation tissue components significantly decreased around the Ti threads spaces (Figure 5D, E, F), while the newly formed bone matrix increased in these regions (Figure 5I). The newly produced bone matrix was deposited immediately adjacent to the bone thread spaces (Figure 4D, D’, E, E’), indicating direct contact between the implant surface and bone after 14 and 21 days. The scattered areas surrounding the Ti thread spaces and bone were left with soft tissue, including connective tissue and bone marrow after 21 days. Furthermore, at the 14^th^ and 21^st^ days after implantation, the peri-implant mucosa exhibited a well-organized connective tissue attachment, composed mainly of fibroblasts and collagen fibers, with small quantities of inflammatory cells.

**Figure 6:**
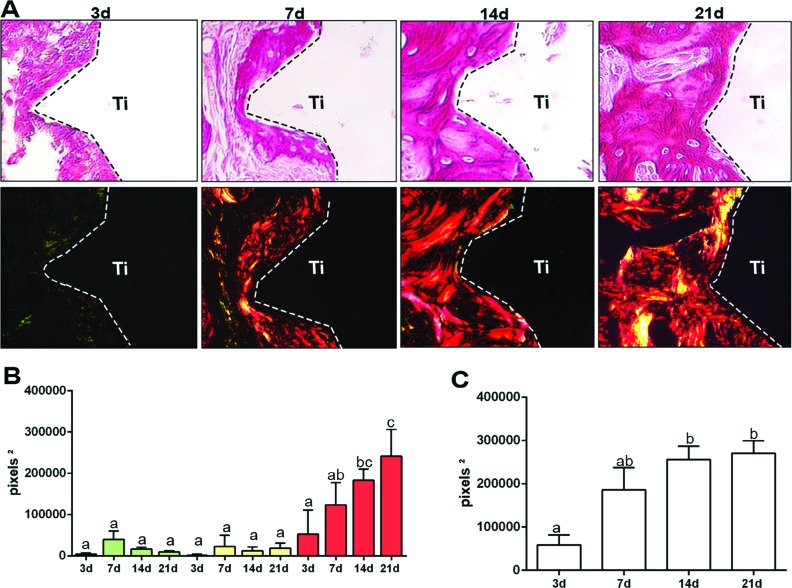
Birefringent fibers by picrosirius-polarization method in the oral osseointegration process. A) Representative sections from oral osseointegration process upon polarized and conventional light, to evaluate collagen fibers maturation along days 3, 7, 14 and 21 post-Ti-screw implantation. As visualized upon polarized light, green birefringence color indicates thin fibers; yellow and red colors at birefringence analysis indicate thick collagen fibers. Original magnification 40x; B-C) Intensity of birefringence measured from Image-analysis software (AxioVision, v. 4.8, Carl Zeiss Microscopy GmbH, Jena, Germany) to identify and quantify (B) area of collagen from each birefringence color (pixels 2) and total area of collagen fibers (pixel2) throughout experimental periods. Results are presented as the mean and SD of pixels2 for each color in the birefringence analysis. Different letters indicate a statistically significant difference (p 0.05) between the different time periods (p<0.05)

For analyzing the maturation dynamics of collagen fibers, we quantified different birefringent collagen fibers (green, yellow and red) from the new bone matrix and initial granulation tissue. A negligible quantity of collagen fibers was found after 3 to 7 days around the Ti threads, emitting birefringence in the green spectrum (i.e. immature and thinner fibers) (Figure 6A). From 7 to 21 days, there was a significant increase in the quantity of total collagen fibers (Figure 6C), as well as in the maturation of the organic matrix, as evidenced by the presence of red spectrum fibers under polarized light (Figure 6A) in parallel with the sequential increase of red intensity pixels area (Figure 6B).

### Gene expression patterns in the osseointegration process

**Figure 7:**
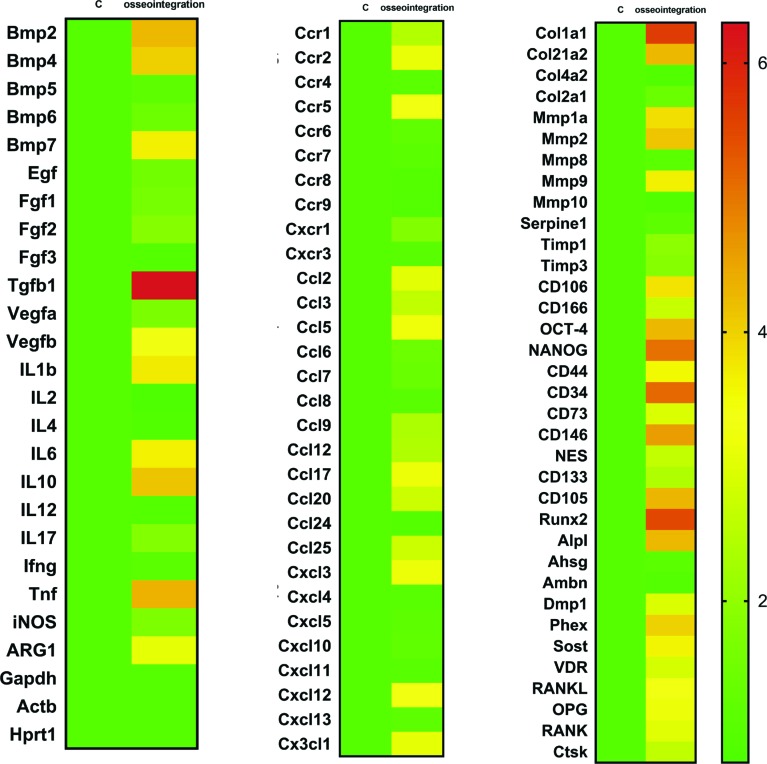
Gene expression patterns in the osseointegration process in C57Bl/6 mice. Molecular analysis of the gene expression patterns in the region of Ti screw implantation was comprised of an initial exploratory analysis by RealTimePCR array, considering a pool of samples from all the experimental time periods (3 d, 7 d, 14 d, 21 d). RealTimePCR array analysis was performed with the VIA7 system (Applied Biosystems Limited, Warrington, Cheshire, UK) using a customized qPCRarray comprised of the major targets from the Osteogenesis, Inflammatory Cytokines & Receptors and Wound Healing panels of the PCRarrayRT2 Profiler (SABiosciences/QIAGEN, Gaithersburg, MD, USA). Results are depicted as the fold increase change (and the standard deviation) in mRNA expression from triplicate measurements in relation to the control samples and normalized by internal housekeeping genes (GAPDH, HPRT, β-actin)

**Figure 8:**
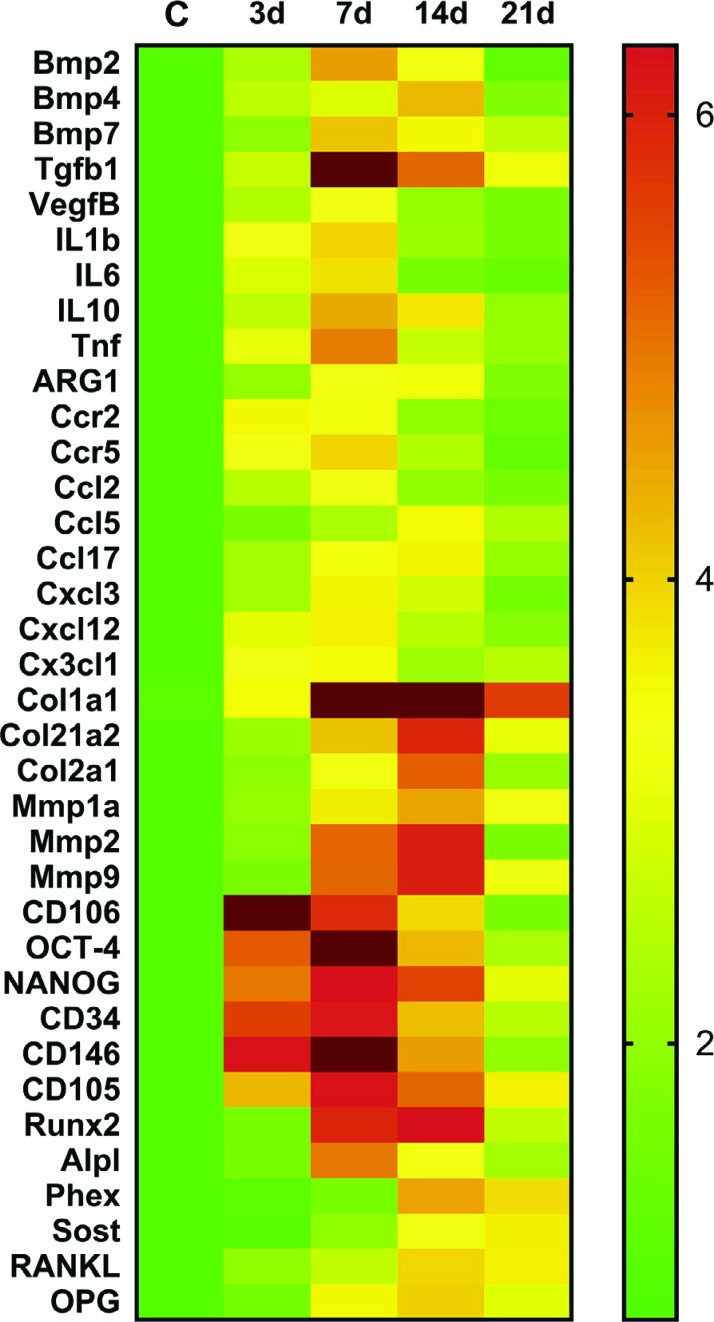
Kinetics of gene expression in the oral osseointegration process in C57Bl/6 mice. RealTimePCR array pooled from of all the experimental time periods was used to identify targets with a significant expression variation for their subsequent analyses in different time points along osseointegration process (0 h, 7 d, 14 d, 21 d). RealTimePCRarray analysis was performed with the VIA7 system (Applied Biosystems, Warrington, UK) using a customized qPCRarray comprised of the major targets from the Osteogenesis, Inflammatory Cytokines & Receptors and Wound Healing panels of the PCRarrayRT2 Profiler (SABiosciences/QIAGEN, Gaithersburg, MD, USA). Results are depicted as the fold increase change (and the standard deviation) in mRNA expression from triplicate measurements in relation to the control samples and normalized by internal housekeeping genes (GAPDH, HPRT, β-actin)

A pool of samples from all periods post-Ti implantation were initially analyzed through an exploratory RealTimePCR array (Figure 7), considering the molecules involved in inflammatory response and bone healing (growth factors; immunological/inflammatory markers; extracellular matrix, MSC and bone markers) to select targets with significant expression in comparison with the control samples. Subsequently, the targets with a significant variation expression in the pooled samples were analyzed according to their kinetics of expression during the experimental periods (Figure 8). Among several growth factors, the BMP2, BMP4, BMP7 molecules and TGFβ1 expression were upregulated during osseointegration in comparison with the control (Figure 7), with a peak of mRNA levels after 7 and 14 days (Figure 8). Considering the immunological markers analyzed (cytokines, chemokines, chemokine receptors and other inflammatory mediators) IL1β, IL6, IL10, TNF, ARG2, CCR2, CCR5, CCL2, CCL5, CCL17, CXCL3, CXCL12, CX3CL1 were positively regulated in the osseointegration process in comparison with the control samples (Figure 7). The kinetics analysis demonstrated that some immunological markers (IL1β, IL6, IL10, TNF, CCR2, CCR5, CCL2, CXCL12, and CX3CL1) were upregulated 3 days after implantation, but all those markers peaked at the 7^th^ day, followed by a gradual decrease in their expression in subsequent experimental periods (Figure 8). Among the extracellular matrix markers, Col1a1, Col21a1, Col2a1, MMP1a, MMP2 and MMP9 were upregulated through the oral osseointegration process in comparison with the control samples (Figure 7). The kinetics analysis demonstrated that Col1a1 peaked after 7 and 14 days, with gradual decrease after 21 days; while Col21a1, Col2a1, MMP1a, MMP2 and MMP9 was upregulated after 7 days and peaked after 14 days with gradual decrease after 21 days. MSC markers CD106, OCT-4, NANOG, CD34, CD146 and positively upregulated CD105 were found in the osseointegration sites, with a peak of expression for CD106 after 3 days, while OCT-4, NANOG, CD34, CD146 and CD105 peaked after 7 days (Figure 8). All these cited MSC markers exhibit significant upregulation after 3, 7 and 14 days, with significant decrease after 21 days (Figure 8). Among the bone markers, upregulated early bone formation markers Runx2 and Alpl, late bone formation markers Phex and Sost, as well as remodeling markers RANKL and OPG were found in the osseointegration sites compared to the control samples (Figure 7). The kinetic analysis demonstrated that Runx2 and OPG had higher mRNA levels mainly after 7 and 14 days, while Alpl peaked after 7 days with gradual decrease after 14 and 21 days. Also in the kinetics analysis, late bone formation markers Phex and Sost were upregulated after 14 and 21 days, and RANKL exhibited higher mRNA levels after 14 and 21 days.

## Discussion

**Figure 9:**
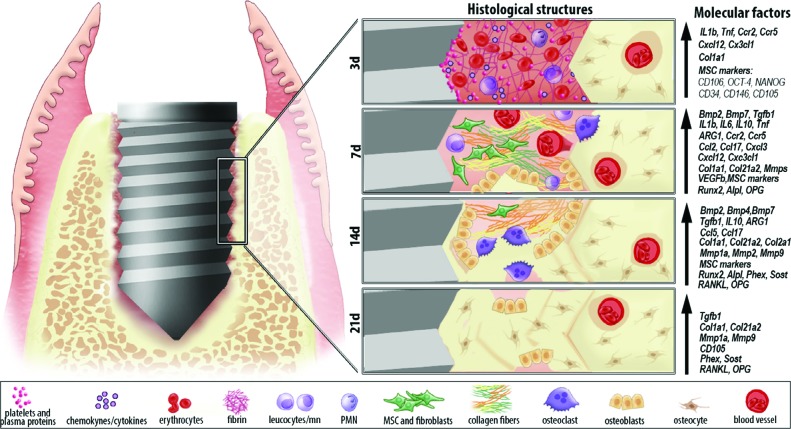
Graphic representation of microscopic and molecular events along oral osseointegration model in mice. Osseointegration process in oral cavity of C57Bl/6 exhibited overlapping phases along 3, 7, 14 and 21 days post Ti implantation. The healing process takes place with an organized blood clot an increased expression of growth factors (TGFb, VEGFb), immunological factors and MSC markers. Subsequently, a provisional granulation tissue is formed, with a high expression of growth factors and earlier osteogenic markers (BMPs, ALP and Runx2). Cytokines (TNF, IL6, IL1, IL10) chemokines (CXCL3, CCL2, CCL5, CC17, CXCL12 and CXC3CL1) and chemokine receptors (CCR2 and CCR5) regulate the infiltration of inflammatory cells and immune response. ARG1 (a M2 marker) is up regulated in implantation sites, indicating a predominance of a M2-type response for macrophages. At late time points (14 and 21 days), bone matrix is significantly increased, also followed by expression of Col1a1 and Col21a2. Remodeling/maturation process of bone is marked by a peak of MMPs, RANKL and OPG expression at 14 days, and an increased presence of osteoclasts. Finally, there is an intimate Ti/bone contact, with an expression of final osteoblast differentiation markers (PHEX, SOST)

Despite the successful clinical application of Ti-based devices, the exact cellular and molecular mechanisms responsible for the osseointegration phenomenon remain unclear, especially considering the immunological pathways involved in this process. In view of the multiple experimental advantages conferred by the use of mice as the experimental host for Ti implantation, in this study we describe the microtomographic, histological/histomorphometric and molecular characterization of an oral maxillary osseointegration model along early (3 and 7 days) to late experimental periods (14 and 21 days) in the oral cavity of C57Bl/6 mice (Figure 9).

While the C57Bl/6 strain was used in a previous study as recipients/hosts of Ti devices in the oral cavity^28^, implants were placed in the maxillary bone after extraction of 3 upper molars. Considering the complex anatomic feature of the upper molars of mice and the potential surgical complications/intercurrences due to exodontic procedures, and the requirement for 2 surgical procedures, we initially performed measurements of palatal bone thickness in C57Bl/6 mice to verify the possibility of implant insertion in the palatal edentulous area. The thicker region of the palatal bone in the edentulous alveolar ridge of C57Bl/6 corresponded to 300 µm, which was considered suitable to receive a miniature Ti implant screw cut at a 1.5 mm length. The implant’s placement in this area, without preceding multiple tooth extraction, was previously reported in CD1 mice, which, due to their increased size, were suitable for the insertion of a 2 mm implant^10^. Additionally, the Ti screw used in this study was based on a conventional Ti6Al4V alloy, with a machined surface without any treatments and/or topography alterations, as demonstrated through SEM and X-ray analysis (Figure 2), in order to characterize the osseointegration process *per se,* as has been frequently used in experimental studies using craniofacial^10^ and long bones^16^ ^,^ ^25^ as osseointegration models.

The surgical procedures used in this study were performed following the same principles and procedures used in Dentistry, to avoid lack of primary stability and overheating. Of all titanium implants with adequate primary stability, 77.78% achieved osseointegration, demonstrated through µCT and histological data (Figures 3 and 4), which is in agreement with the success rates previously described in a similar model performed in CD1mice (74% of osseointegration after 21 days)^10^. Additionally, the 5 implants which exhibited failure after 14 and 21 days had a fibrous connective tissue surrounding the Ti screw area with no signs of infection. Osseointegration failure in these specimens could possibly be a result of loosening of the primary stability in the first few days post-Ti implantation.

Initially, our histological characterization demonstrated that blood is the first biological element in contact with the Ti surface, evidenced by the formation of a highly organized clot in contact with the Ti threads and native bone after 3 days (Figures 4B and B´) as also observed in larger models in rats^27^, where blood components, such as the fibrin network, provide a structural support for initial cell adhesion and migration toward the implant’s surface^28^. Indeed, at the early stages, a protein adsorption layer is created on the Ti surfaces, constituted mainly by blood molecules, platelets and plasma fibronectin, as also demonstrated by *in vitro* studies^29^ where the presence of plasma fibronectin on the Ti surface supports the first events of osteogenesis. It is interesting to note that, theoretically, this first protein layer on the Ti surfaces also contains molecules required for regulation of the subsequent steps that will lead to osseointegration^30^, such as growth factors and immunologic mediators, which orchestrates bone formation in the peri implant space^27^. In agreement with this, our molecular data demonstrated an upregulation of TGFb1 and CXCL12 in the early stages after Ti implantation (Figure 8), which were also observed in the early stages of oral osseointegration in rats^27^. In the osseointegration context, TGFb1 and CXCL12 have been shown to enable the migration of mesenchymal osteoprogenitor cells on the implant’s surface and threads spaces^2^ ^,^ ^31^. Accordingly, MSC are among the first cells to migrate to the Ti surface^31^, and in fact, several MSC markers (CD106, OCT-4, NANOG, CD34, CD146 and CD105) also exhibited early upregulation post-Ti implantation (Figure 8).

Concurrently with the early upregulation of the MSCs markers, a provisional extracellular matrix is formed and gradually evolves into a highly vascularized granulation tissue (Figures 4 and 5), which will provide further support for cell migration and differentiation. A similar response was observed in peri-implant sites in mice^10^ ^,^ ^22^ and rats^27^, but the presence of biomaterials was associated with delayed healing dynamics compared to alveolar intramembranous bone healing in the absence of biomaterials^9^ ^,^ ^10^. Indeed, the earlier granulation tissue formed in the space between the Ti threads and remaining bone works as a preosteoblastic supportive connective tissue^10^ ^,^ ^22^, as evidenced in this study by an increased area density of blood vessels (Figure 5D), fibroblasts (Figure 5E) and osteoblasts (Figure 5G) after 7 days in the implantation sites, with upregulation of angiogenic (VEGFb) and earlier of osteogenic markers (BMP2,4 and 7, ALP and Runx2) (Figure 8). Indeed, BMPs (BMP2, BMP4 and BMP7) are key factors related to the commitment of MSC into osteoblast fate during physiological osteogenesis^32^, bone repair^10^ and osseointegration^33^, since BMPs can stimulate transcription factor RUNX2^32^. It should be noted that RUNX-2 directly binds itself to enhancer regions of osteoblast-specific genes, such as the earlier matrix mineralization ALP^34^, which is also in agreement with our findings.

Also in these earlier stages of osseointegration, the immune/inflammatory response is triggered at the Ti/host interface, which integrates the key molecular events for determining the success or failure of osseointegration^3^ ^,^ ^35^. Indeed, in this study the area density of inflammatory cells peaked in the earlier periods of the osseointegration process, in parallel with an upregulation of a variety of immunological factors involved in leukocyte migration, such as pro-inflammatory cytokines (TNF, IL6, IL1) and monocytes/macrophages chemoattractants (i.e. chemokines CXCL3, CCL2, CCL5, CC17, CXCL12 and CXC3CL1) and chemokine receptors (CCR2 and CCR5), were highly expressed in the sites of implantation (Figures 7 and 8). In agreement with these findings, an early molecular assessment of the osseointegration process in humans revealed a similar pattern of expression of chemokines and interleukins in the early periods post-Ti implantation^30^, which was also observed in rats^27^, reinforcing the validity of the mouse model due to the similar inflammatory response pattern. It should be noted that while TNF, IL6 and IL1 comprise part of a macrophage cytokine portfolio, CCR2 and CCR5 are involved mainly in monocytes/macrophages migration for wound healing, suggesting an important involvement of macrophages with the oral regenerative processes^35^.

Indeed, in addition to the classical role of macrophages on debris clearance after injury, these cells are key regulators of inflammatory and regenerative processes, by releasing different mediators in response to the state of polarization towards the M1 (inflammatory) or M2 (reparative) phenotype, and orchestrating the outcomes of inflammation and bone healing^36^. Interestingly, it has been proposed that activation of these cells into M1 and M2 macrophages is a crucial step for orchestrating a foreign body reaction (FBR) after implantation of biomaterial and also to determine the equilibrium between osteogenic factors/cells and osteolytic factors/cells around the Ti implant after osseointegration^2^ ^,^ ^3^. In this study, while iNOS (a M1 marker) expression remained low at the osseointegration sites, ARG1 (a M2 marker) was upregulated after Ti implantation, indicating a predominance of a M2-type response. Indeed, in enhanced osseointegration models observed in long bones in rats, the upregulation of ARG1 and downregulation of iNOS are correlated with a high proportion of M2 macrophages and beneficial bone healing around the Ti surfaces^37^. Accordingly, a marked-up regulation of reparative/regulatory M2-type macrophages is also observed after Ti implant placement in humans^30^. Indeed, the M2-type response has been suggested to be critical to wound healing outcomes for expressing several pro-resolutive molecules, including ARG1, IL10 and TGFb1^38^. These data are also compatible with the transitory nature of the inflammatory infiltrate surrounding the Ti surface, which showed a gradual decrease over time in this study (Figure 4B, C, D and Figure 5).

Following the resolution of inflammation (Figure 4D), while the expression of inflammatory factors and density of inflammatory infiltrate tend to decrease over time post- implantation, the expression of osteogenic factors and ECM components were gradually increased, in agreement with previous findings in rats^27^. In line with the events of intramembranous bone repair, the granulation tissue is directly replaced by bone over time (Figures 3 and 4), as also previously reported in other animal models of oral osseointegration ^10^ ^,^ ^27^, while Ti osseointegration in long bones is dependent on the formation of hypertrophic cartilage^15^. As the density area of the primary bone matrix significantly increased after 14 days, also followed by expression of Col1a1 and Col21a2 and a gradual maturation of collagen fibers detected through birefringence analysis (Figure 6), there was a remarkable remodeling process, evidenced by peaks corresponding to MMPs (MMP1, MMP2 and MMP9), RANKL and OPG, and also an increased area density of osteoclasts (Figure 5H). As also demonstrated in other models^28^ ^,^ ^30^, all these events will collectively determine bone quality and influence the mechanical properties of osseointegration^37^. Indeed, the quality of osseointegration is dependent on a highly organized bone matrix and its ECM components, in which collagen plays a crucial role^38^.

Consequently, in late stages, there was intimate bone contact over the Ti threads, associated with the expression of several bone markers typical of final osteoblast differentiation (PHEX, SOST)^9^. It should be noted that the maximum amount of osseointegration was achieved in C57Bl/6 mice at the 21^st^ day, with an average 42.12±3.01% mineralized bone matrix (BV/TV) detected around the Ti threads via microCT analysis (Figure 3), and also 87% of the total collagen content detected through birefringence analysis being red spectrum collagen fibers (Figure 6), possibly an indicative of well-organized collagen fiber bundles^9^ ^,^ ^10^. Interestingly, in the complementary histomorphometry analysis, the percentage of bone matrix around/and in contact with the Ti threads represented a an average 81.03±3.87% density area, which is in agreement with histological investigations of Ti dental implants placed in humans, where the bone area in the individual threads reached 81.8% in average^38^. However, even 60% of histological bone-to-implant contact is considered as enough osseointegration for successful implants in humans for up to 17 years^3^.

## Conclusions

In summary, this study originally demonstrated a unique molecular view of the kinetics of osseointegration, evidencing elements that could be responsible for orchestrating cell migration, proliferation, ECM deposition and maturation, angiogenesis, bone formation and remodeling at the bone-implant interface in parallel with a novel histological, birefringence and μCT analysis (Figure 9). Considering all these observations and comparing with previous descriptions of osseointegration, this C57Bl/6 mice oral osseointegration model would be a suitable tool for the assessment of biological events associated with the osseointegration process.
